# Supplementary material for: Improving oral health in people with severe mental illness (SMI): A systematic review
Source: PLoS One. 2021 Dec 1;16(12):e0260766. doi: 10.1371/journal.pone.0260766 (PMC8635332; doi:10.1371/journal.pone.0260766)
Supplement: S2 File — (DOCX) [file pone.0260766.s002.docx]

**S2 File. Search strategy for EMBASE**

1. exp Schizophrenia/

2. exp Paranoid Disorders/

3. exp Bipolar Disorder/

4. schizo*.mp.

5. hebephreni*.mp.

6. psychosis.mp.

7. psychotic*.mp.

8. psychoses.mp.

9. ((chronic* or sever*) adj2 mental* adj2 (ill* or disorder*)).mp.

10. ((bipolar or delusion*) adj disorder).mp.

11. 1 OR 2 OR 3 OR 4 OR 5 OR 6 OR 7 OR 8 OR 9 OR 10

12. (toothbrush* or tooth-brush* or floss* or "chewing stick*" or "wood stick*" or toothpick*).mp.

13. ((dental or oral or mouth or interdental or interproximal or tooth or teeth or orthodontic or denture* or brace* or bracket*) adj3 (irrigat* or clean* or brush*)).mp

14. (dentifrice* or mouthwash* or mouthrins* or mouth-wash* or mouth-rins*).mp.

15 (plaque* adj5 (remov* or control*)).mp.

16. Dental health education/

17. ((health* adj3 promot*) and (dental or teeth or tooth or mouth or periodont* or gingival* or "oral health")).mp.

18. ((oral or dental) adj2 (hygiene or care)).mp.

19. ((mouth or teeth or tooth) adj3 care).mp.

20. Mouth hygiene/

21. exp Periodontal disease/

22. exp Periodontics/

23. (periodont* or gingiv*).mp.

1. (caries or carious).mp.
2. exp dental caries/
3. Tooth plaque/

27. ("dental plaque index" or "dental plaque indices" or "DMF* index" or "DMF indices" or "dmf* index" or "dmf* indices" or "periodontal index" or "periodontal indices" or "oral hygiene index" or "oral hygiene indices" or "gingival index").mp.

28. ((access* or attend* or visit*) and ((dent* adj2 (service or practice)) or (dentist* or "dental practitioner"))).mp

29. ("OHRQoL" or ((quality adj2 life) and ((oral or dental) adj2 health))).mp

30. 12 OR 13 OR 14 OR 15 OR 16 OR 17 OR 18 OR 19 OR 20 OR 21 OR 22 OR 23 OR 24 OR 25 OR 26 OR 27 OR 28 OR 29

31. 11 AND 30

**Search strategy for Medline**

1. exp Schizophrenia/

2. exp Paranoid Disorders/

3. exp Bipolar Disorder/

4. schizo*.mp.

5. hebephreni*.mp.

6. psychosis.mp.

7. psychotic*.mp.

8. psychoses.mp.

9. ((chronic* or sever*) adj2 mental* adj2 (ill* or disorder*)).mp.

10. ((bipolar or delusion*) adj disorder).mp.

11. 1 OR 2 OR 3 OR 4 OR 5 OR 6 OR 7 OR 8 OR 9 OR 10

12. (toothbrush* or tooth-brush* or floss* or "chewing stick*" or "wood stick*" or toothpick*).mp.

13. ((dental or oral or mouth or interdental or interproximal or tooth or teeth or orthodontic or denture* or brace* or bracket*) adj3 (irrigat* or clean* or brush*)).mp

14. (dentifrice* or mouthwash* or mouthrins* or mouth-wash* or mouth-rins*).mp.

15 (plaque* adj5 (remov* or control*)).mp.

16. Dental health education/

17. ((health* adj3 promot*) and (dental or teeth or tooth or mouth or periodont* or gingival* or "oral health")).mp.

18. ((oral or dental) adj2 (hygiene or care)).mp.

19. ((mouth or teeth or tooth) adj3 care).mp.

20. Oral hygiene/

21. exp Periodontal disease/

22. exp Periodontics/

23. (periodont* or gingiv*).mp.

24. (caries or carious).mp.

25. exp dental caries/

26. Dental plaque/

1. ("dental plaque index" or "dental plaque indices" or "DMF* index" or "DMF indices" or "dmf* index" or "dmf* indices" or "periodontal index" or "periodontal indices" or "oral hygiene index" or "oral hygiene indices" or "gingival index").mp.

28. ((access* or attend* or visit*) and ((dent* adj2 (service or practice)) or (dentist* or "dental practitioner"))).mp

29. ("OHRQoL" or ((quality adj2 life) and ((oral or dental) adj2 health))).mp

30. 12 OR 13 OR 14 OR 15 OR 16 OR 17 OR 18 OR 19 OR 20 OR 21 OR 22 OR 23 OR 24 OR 25 OR 26 OR 27 OR 28 OR 29

31. 11 AND 30
