# Supplementary material for: Improving oral health in people with severe mental illness (SMI): A systematic review
Source: PLoS One. 2021 Dec 1;16(12):e0260766. doi: 10.1371/journal.pone.0260766 (PMC8635332; doi:10.1371/journal.pone.0260766)
Supplement: Attachment — Submitted filename: Dr Teng Plos One comment.doc [file pone.0260766.s003.doc]

Oral health improvement is important for persons with severe mental illness. This review article is well structured and the manuscript content is detailed. Furthermore, it provides more insight for practice and can facilitate further research to enhance oral hygiene for this disadvantaged patient group. However, several points must be addressed before this manuscript can be published.

1. The authors reviewed relevant articles that are published prior to May 2020. I think that the following two articles are relevant and recommend that they be included in the literature review:

(1) Mun, S.J. et al. Reduction in dental plaque in patients with mental disorders through the dental hygiene care programme. Int J Dent Hygiene 2013;12(2):133–40.

(2) Yoshii H, et al. Effects of an educational intervention on oral hygiene and self-care among people with mental illness in Japan: a longitudinal study. BMC Oral Health 2017; 17:81.

Please consider adding these two articles into the literature review.

2. Moreover, if the authors had extended the scope of the literature review to the end of 2020, the review could have included an additional cluster randomized controlled trial (RCT) published by Kuo et al. (Effectiveness of oral health promotion program for persons with severe mental illness: a cluster randomized controlled study. BMC Oral Health 2020; 20:290).

I believe that if the authors had included the abovementioned three articles, the manuscript would be more comprehensive.

3. In the 2nd paragraph of the discussion section, the authors mentioned that oral health education might increase individual awareness of personal oral health in a negative manner. However, Teng, P.R. et al. (Gen Hosp Psychiatry 2011; 33: 253-259) showed that the neglect of oral health in persons with severe mental illness is in part due to the lack of awareness. Specifically, nearly 40% of the psychiatric inpatients rated their oral health as good despite the presence of periodontal pockets. Therefore, a dissatisfactory perception of oral health in an intervention group might not be negative because it could enhance their awareness of poor oral health and motivate them to take actions to improve oral hygiene.
