# Supplementary material for: Improving oral health in people with severe mental illness (SMI): A systematic review
Source: PLoS One. 2021 Dec 1;16(12):e0260766. doi: 10.1371/journal.pone.0260766 (PMC8635332; doi:10.1371/journal.pone.0260766)
Supplement: Attachment — Submitted filename: Response to reviewers.docx [file pone.0260766.s004.docx]

University of York

Heslington

York

YO10 5DD

27^th^ August 2021

Dear Dr Lalloo,

Thank you for considering our study for publication in *PLOS ONE* and many thanks to the reviewers for their constructive feedback, which has been incredibly valuable in improving the manuscript. In response to the feedback, we have summarised the comments and our responses in the table below.

| **Comment** | **Response** |
| --- | --- |
| Please ensure that your manuscript meets PLOS ONE's style requirements, including those for file naming | The title, headings, tables and file names have been amended to ensure that the style requirements have been met. |
| We note that you have indicated that data from this study are available upon request. | As all key data is included in the manuscript, this section has now been amended to state *“*All relevant data are within the manuscript and its Supporting Information files” |
| **Reviewer One** | |
| It is suggested that the participant, intervention, comparison, and outcome (PICO) framework be used to formulate the research focused question. In that way, readers will have a more clear idea of what to expect from the paper. | This has now been clarified in this format (pages 6 and 7) |
| Page5, L 88. What authors mean by adults? What is the age range? | This has now been clarified to state adults are defined as aged over 18 (page 6, L 99 – clean copy of manuscript) |
| Page 5, L 90. It is suggested that authors describe more in detail the other psychotic disorder since readers in the field of dentistry may not be familiar with them. | Thank you for this insight. As the term “psychotic disorders” encompasses many different potential diagnoses, these have not all been listed, but the term has been clarified and an example provided (page 6, L 101-102). |
| Authors need to clearly define inclusion and exclusion criteria under separate subheadings so it would be easier for readers to follow. | The inclusion and exclusion criteria have now been separated for clarity (page 7, L 119-122) |
| Page 5 L 92. What do authors mean by dual diagnosis exactly? SMI and another psychological disorder? | This has been clarified to now state “dual psychiatric diagnosis” (page 6, L 105) |
| Authors need to mention the exact month for their systemic search in 2020 for each database. | This has now been included (page 7 L 130). |
| Why 90% of the screening of titles and abstracts were done by one author? It’s not a common approach in conducting systematic reviews and usually 2 authors screen all the records. Moreover, does 100% agreement between the two authors, when piloting for 10% of the records, refer to those papers that were excluded, included, or both? If it only refers to excluded papers, it can be a source of bias. | The search has been repeated with 100% of title and abstract screening undertaken independently by two reviewers to reduce the risk of bias (page 8, L135-137) |
| Only seven papers were included in this review which consequently prevents authors from conducting meta-analysis due to heterogeneity. By a quick search only in PubMed, the following papers were found that possibly could have been included in this review. | The search has now been repeated, meaning that the review now has 12 studies in total. Thank you for taking the time to check for eligible studies, however some of the suggested studies did not meet the study criteria (e.g. study protocol, qualitative study), however one of the suggested studies that had been published after the initial search has now been included in the review. |
| Page 8 L 162. Only one reference in the bracket was mentioned which seems to be by mistake and the authors intended to cite all the included papers? | Thank you – citations of all the studies have now been included, and the additional citation at the end of this section is to reference the PRISMA flowchart (page 10 L 182 and 185). |
| It is suggested that the excluded papers in the full-text screening phase will be cited as well. | These have now been included (page 10 L 183-185) |
| It is suggested that interventions be mentioned in tables 2 and 3 as well. | These have now been included |
| Please cite or mention the names and authors of the related papers in Table 4. | Citations have now been incorporated into Table 4 |
| Discussion: After including any possible papers this section needs to be revised. The categorization of this section seems to be fine, but it is suggested that authors focus more on synthesizing the results and providing a comprehensive interpretation of them. Some paragraphs are the repetition of the results and lengthy and can be better summarized. | Thank you for the helpful feedback. In addition to incorporating the results from the five additional studies, the elements of the discussion that are more repetitive (particularly in relation to the results of studies) have been removed. |
| **Reviewer Two** | |
| Your introduction section establishes the importance of the topic, but some of the concepts and dental terminology are not clearly explained. | The clinical terms and concepts in the introduction have been explained or have been replaced with clearer language. |
| Your study lacked a clear research question, and the inclusion criteria were very broad, so it was unsurprising that there was too much heterogeneity between studies to conduct a meta-analysis (synthesis would require more commonality between studies). | The research question has been clarified within the introduction (page 5, L 87-89) and through the inclusion criteria presented using the PICO framework (pages 6 and 7). We appreciate that the inclusion criteria were very broad, however we have tried to clarify the rationale for this within the introduction (page 5, L 78-86). |
| Despite such broad criteria, only seven studies were included in the analysis, and two of those were published in the 1970s. The results were presented more like a simple descriptive summary of studies, which minimises the usefulness of the findings. Unfortunately, I do not think this study contributes valuable new findings for practice or research and am therefore not recommending it be accepted for publication. | The authors have repeated the search for the review, which now includes five additional studies. Although we acknowledge that it would be preferable to include a meta-analysis we felt that the included studies were too heterogeneous to conduct a meaningful meta-analysis.  We believe that this narrative review provides a valuable summary of the evidence around oral health interventions in this population. |
| In considering the quality of the writing, I found myself confused on occasion as some of the comments were rather vague or unclear, and the narrative of the manuscript did not flow well. There were some structural issues with paragraphs and sentences. Try to avoid anthropomorphisms e.g. ‘three studies had some concerns…’ These problems with structure and writing conventions can be easily rectified. | Thank you for the constructive feedback. The narrative has now been amended to provide more clarity and flow, and reduce repetition (for example within the introduction and discussion). The risk of bias assessments have been simplified to avoid unclear narrative and anthropomorphisms, thank you for noting this. |
| **Reviewer Three** | |
| The authors reviewed relevant articles that are published prior to May 2020. I think that the following two articles are relevant and recommend that they be included in the literature review: | Thank you very much for the helpful advice. The article by Mun et al 2014 has been included in this revision of the review, however the article by Yoshii did not meet the inclusion criteria, so was not included. |
| Moreover, if the authors had extended the scope of the literature review to the end of 2020, the review could have included an additional cluster randomized controlled trial (RCT) published by Kuo et al. (Effectiveness of oral health promotion program for persons with severe mental illness: a cluster randomized controlled study. BMC Oral Health 2020; 20:290). I believe that if the authors had included the abovementioned three articles, the manuscript would be more comprehensive. | Thank you very much for this suggestion. The search has been updated and the review now includes five additional articles (Mun and Kuo et al and three others). |
| In the 2^nd^ paragraph of the discussion section, the authors mentioned that oral health education might increase individual awareness of personal oral health in a negative manner. However, Teng, P.R. et al. (Gen Hosp Psychiatry 2011; 33: 253-259) showed that the neglect of oral health in persons with severe mental illness is in part due to the lack of awareness. Specifically, nearly 40% of the psychiatric inpatients rated their oral health as good despite the presence of periodontal pockets. Therefore, a dissatisfactory perception of oral health in an intervention group might not be negative because it could enhance their awareness of poor oral health and motivate them to take actions to improve oral hygiene. | Thank you for this useful suggestion. These research findings have now been incorporated into the discussion with reference (page 29-30, L 444-446 on clean version of manuscript) |

If you have any further queries, please do not hesitate to get in touch.

Yours Sincerely

Alex Macnamara
