# Supplementary material for: Improving oral health in people with severe mental illness (SMI): A systematic review
Source: PLoS One. 2021 Dec 1;16(12):e0260766. doi: 10.1371/journal.pone.0260766 (PMC8635332; doi:10.1371/journal.pone.0260766)
Supplement: Attachment — Submitted filename: Response to reviewers.docx [file pone.0260766.s005.docx]

University of York

Heslington

York

YO10 5DD

27^th^ August 2021

Dear Dr Lalloo,

Thank you again for considering our study for publication in *PLOS ONE* and many thanks to the reviewers for their constructive feedback, which has been incredibly valuable in improving the manuscript. In response to the additional feedback on the revised manuscript, we have summarised the comments and our responses in the table below.

| **Comment** | **Response** |
| --- | --- |
| Please review your reference list to ensure that it is complete and correct | Reference 1 URL updated.  Reference 7 has been updated to include the URL.  Reference 10 URL updated.  Duplicate reference noted (5 and 11) – duplicate removed – reference 5 now only reference to this site.  Reference 17 updated to correct author names.  Reference 18 URL updated.  Reference 23 URL updated.  Reference 36 URL updated.  Reference 42 URL updated.  Reference 44 URL updated.  Reference 48 URL updated.  Reference 85 URL updated. |
| **Reviewer One** | |
| When reporting the results using PICO make sure to mention in advance in the text in the Method section and used the appropriate reference. Also, the main research question should be formulated before going into detail about categories. Please see similar papers in the literature to get familiar with the proper reporting format. | Thank you for this helpful suggestion. Following the research aim at the end of the introduction (page 5, lines 88-90 – clean copy), the methods section has now been updated to reflect the use of the PICO framework with the relevant reference (page 6, lines 98-100). |
| Please use the subheading “Inclusion Criteria” instead of “Study Design and Language” | This has now been changed as suggested (Page 7, line 117) |
| In the response letter, the authors mentioned that qualitative studies were not considered eligible. If this is the case, please mentioned it in your exclusion criteria. | This has now been included for clarity, thank you. Please see page 7 line 125. |
| Based on the Prisma flowchart, out of the 5,330 screened records, only 16 studies were considered eligible for full-text reading which is proportionally a low rate implying a strict screening process. As a suggestion, please go through those papers you find closely related and evaluate their eligibility based on the full-text reading to make sure no study is missing. If the number of excluded is fairly small (10 or under), please use a table or explain in more detail the reason for exclusion for each paper specifically. | Thank you for noting this.  The 5,330 records encompassed both duplicate records and conference abstracts, meaning that in total 1,465 records from the database search were screened, with 16 identified for full text screening. The search was also broad as it aimed to encompass the wide range of potential interventions and outcomes, therefore we were anticipating that there would be a number of unrelated articles.  The reasons for exclusion from the full text screening are listed with references (page 10, lines 186-189). |
| Regarding the quality of writing and academic style, some editing is needed since the flow is lost in some sections. | Changes have been made to the writing in order to improve the flow and ensure clarity and consistency. |
| Reviewer #3: In the section of abstract, "There is a currently a lack of evidence to suggest..." The double "a" should be deleted to one "a". | Thank you for making us aware of this, this has now been amended to read "There is currently a lack of evidence to suggest..." (Page 2 – line 23) |

If you have any further queries, please do not hesitate to get in touch.

Yours Sincerely

Alex Macnamara
