# Supplementary material for: Improving oral health in people with severe mental illness (SMI): A systematic review
Source: PLoS One. 2021 Dec 1;16(12):e0260766. doi: 10.1371/journal.pone.0260766 (PMC8635332; doi:10.1371/journal.pone.0260766)
Supplement: Attachment — Submitted filename: Response to reviewers.docx [file pone.0260766.s006.docx]

University of York

Heslington

York

YO10 5DD

9^th^ November 2021

Dear Dr Lalloo,

Thank you again for considering our study for publication in *PLOS ONE*. We would once again like to thank the reviewers for taking the time to review this manuscript and for their input and feedback, which has been of such help and value in improving this manuscript. In response to the further feedback on the revised manuscript, we have summarised the comments and our responses in the table below.

| **Comment** | **Response** |
| --- | --- |
| **Reviewer One** | |
| “Some of the comments have been address in this versin, but authors need to excersise more caution when reporting the results and findings so that the results will be homogeneous throughout the manuscript. As mentioned before, the Prisma flowchart has not been revised. It clearly mentions in your Prisma flowchart that 5,333 records have been obtained after the dublicates have been removed, and the whole screening process is based on the 5,330 record!  Also in the reposnse letter authors mentioned 1,465 unique record were identified (if this is the case, this number should be reported in the abstract as well). However in the manuscript this number is 1,462! and again 2,073 was mentioned instead of 5,330 in the text for numbers of records before excluding the duplicates! In my opinion, the authors need to address these controversies since for potential readers it will be not possible to know what approch the authors took to select the final inluded studies.” | Thank you very much for your feedback on this reporting of the search strategy  To avoid confusion, we have amended the flow chart so that it only includes records identified from the database and grey literature search. We now only describe the hand searching of conference proceedings in the text (as this did not identify any records). We hope this much more clearly reports the screening process. |

If you have any further queries, please do not hesitate to get in touch.

Yours Sincerely

Alex Macnamara
